# Early Thyroid Volume Reduction in Subacute Thyroiditis Can be a Potential Indicator for Hypothyroidism

**DOI:** 10.3389/fendo.2022.888018

**Published:** 2022-05-30

**Authors:** Ji Yong Park, Wonsuk Choi, A Ram Hong, Jee Hee Yoon, Hee Kyung Kim, Ho-Cheol Kang

**Affiliations:** Division of Endocrinology and Metabolism, Department of Internal Medicine, Chonnam National University Medical School, Gwangju, South Korea

**Keywords:** subacute thyroiditis, thyroid volume, hypothyroid phase, thyroid ultrasonography, hypothyroidism

## Abstract

**Background:**

Thyroid volume has been reported to decrease significantly after episodes of subacute thyroiditis (SAT); however, the relationship between thyroid volume and hypothyroidism remains unclear. This study assessed the association between thyroid volume changes and the hypothyroid phase in patients with SAT, a condition that can progress to persistent hypothyroidism.

**Methods:**

This retrospective study evaluated 37 patients diagnosed with SAT at the Department of Endocrinology and Metabolism of Chonnam National University Hwasun Hospital (CNUHH) between 2016 and 2021. Since we could not determine the clinical characteristics of patients with SAT before their episodes, 120 healthy individuals who underwent thyroid ultrasonography during regular check-ups from 2019 to 2021 at CNUHH were selected for comparison. Subgroup analyses were performed on patients with SAT with and without the hypothyroid phase during their clinical course.

**Results:**

Thyroid volume was significantly greater in SAT patients at the first visit than in controls (p<0.05), and it decreased constantly throughout the follow-up period. Subgroup analysis showed that the initial thyroid volumes were similar in patients with SAT with and without the hypothyroid phase. However, SAT patients with the hypothyroid phase had significantly smaller thyroid volumes at the 1 month (p=0.025) and 3 month (p=0.006) follow-up visits. The reduction rate of the thyroid volume was significantly different within the first month (p=0.009).

**Conclusion:**

A greater reduction in thyroid volume in SAT patients within 1 month of episode had a higher chance of developing a subsequent hypothyroid phase, which can lead to persistent hypothyroidism. Serial thyroid ultrasonography in patients with SAT, especially within the first month, may help in predicting the disease course of SAT.

## Introduction

Subacute thyroiditis (SAT) is an inflammatory thyroid disease presenting with various symptoms, including anterior neck pain and tenderness on physical examination, an enlarged hard thyroid gland, fever, chills, weight loss, palpitation, and/or dyspnea (1, 2). SAT is more prevalent in women than in men and varies by season, and its clinical characteristics are dependent on certain HLA subtypes (3–7). In addition to typical clinical symptoms, SAT is characterized by abnormalities in thyroid function tests, elevation in erythrocyte sedimentation rate (ESR) and/or C-reactive protein (CRP), hypoechoic involvement showing low vascularity on thyroid ultrasonography, and decreased radioactive iodine uptake (RAIU) on scintigraphy and/or fine-needle aspiration biopsy (FNAB) compatible with SAT (1, 2, 8–12). Nonsteroidal anti-inflammatory drugs (NSAIDs) and corticosteroids, which alleviate clinical symptoms and reduce inflammation, are recommended treatments for patients with SAT (2, 13). American Thyroid Association (ATA) guidelines state that approximately 50% of patients with SAT present with thyrotoxicosis, which is caused by a release of pre-formed thyroid hormones, and approximately 30% present in a hypothyroid phase (2). Most patients with SAT recover to a euthyroid state within the first year, whereas 5% to 15% progress to developing persistent hypothyroidism and requiring levothyroxine treatment (1, 2).

Preceding viral infections or inflammatory reactions to viruses are considered the major environmental factors associated with SAT (14, 15). In the era of the COVID-19 pandemic, interest in SAT has been increasing as it can present as one of the extrapulmonary manifestations of SARS-CoV-2 infection or as a side effect of SARS-CoV-2 vaccinations (16–18).

Studies evaluating the association between thyroid volume and dysfunction in SAT have yielded inconsistent results (19–21). Therefore, the present study explored the relationship between time-dependent changes in thyroid volume and thyroid dysfunction in patients with SAT, as well as determined whether early changes in thyroid volume are indicators of a hypothyroid phase, which can progress to permanent hypothyroidism.

## Materials and Methods

### Patients and Treatment

After retrospectively reviewing 113 patients, who visited the department of Endocrinology and Metabolism at Chonnam National University Hwasun Hospital, with an impression of SAT from 2016 to 2021, 37 patients were included in this study. In total, 19 patients with normal ESR, 40 patients who did not have serial thyroid ultrasonography examinations, and 17 patients who were lost to follow-up before SAT resolution were excluded. All 37 patients were diagnosed with SAT before initiating treatment, based on the modified criteria proposed by Stasiak et al., i.e., if the patients met all the main criteria (elevated ESR and hypoechoic lesions with low vascularity and blurred margin on ultrasonography) and at least one of the additional criteria (hard enlarged thyroid, pain, and tenderness when performing physical examinations of the thyroid gland, laboratory tests showing thyrotoxicosis, low radioiodine uptake, or typical result for SAT by fine needle aspiration biopsy) (2, 12, 22). All patients were initially treated with prednisolone ranging from 15 to 30 mg/day, depending on the patient’s clinical status. SAT resolution was confirmed when the patients’ symptoms were relieved, laboratory tests were normalized, and hypoechoic lesions had disappeared on follow-up ultrasonography after the end of the treatment. The patients visited the clinic every month with laboratory tests (thyroid function test and inflammatory markers) in the acute phase, and follow-up intervals increased after resolution. The intervals of thyroid ultrasonography performed were as follows: initial visit, 1 month after the initial visit, 3 months after the initial visit, and longer follow-up periods if the patient’s thyroid function tests were not normalized or their symptoms persisted. Patients with subclinical hypothyroidism undergo follow-up thyroid function tests without hormone replacement, as most patients recover to a euthyroid state within 1 year (2). Patients who had severe clinical hypothyroidism during follow-up were prescribed levothyroxine; two patients presented with persistent hypothyroidism requiring thyroid hormone replacement. Since the characteristics of patients with SAT could not be verified before their SAT episodes, 120 age- and sex-matched healthy controls who underwent thyroid ultrasonography during regular medical check-ups from 2019 to 2021 at Chonnam National University Hwasun Hospital were recruited for comparison. After meticulous review, all controls were confirmed to have no nodules or nodules <5 mm in diameter on ultrasonography evaluation.

### Thyroid Ultrasonography and Thyroid Volumes

Thyroid ultrasonography examinations were performed by highly experienced endocrinologists using the ACUSON S2000 or ACUSON Sequoia Ultrasound System (Siemens Healthineers, Germany). Follow-up ultrasonography was performed by the same physician who performed the initial examination. All ultrasound images were reviewed by a clinician (JY Park).

Thyroid volume was calculated as:


$$Thyroid\;volume\;\left(cm^{3}\right)\text{ = }0.52\;\left(or\;\pi /6\right)\;\times \left(length\;\left[cm\right]\;\times width\;\left[cm\right]\;\times depth\;\left[cm\right]\right)$$

### Laboratory Tests

Complete blood count (CBC), including white blood cell (WBC) count, neutrophil count, lymphocyte count, hemoglobin concentration, platelet count, and mean platelet volume (MPV), were analyzed using an XE-2100 system (Sysmex Corporation, Japan). The concentrations of thyroid stimulating hormone (TSH), free thyroxine (FT4), total triiodothyronine (TT3), anti-thyroid peroxidase antibody (anti-TPO), and anti-thyroglobulin antibody (anti-Tg) were measured using a Cobas e601 analyzer (Roche Diagnostics, Mennheim, Germany). CRP concentrations were measured using Labospect 008AS (Hitachi, Japan), and ESR was analyzed using TEST 1 (Alifax, Padova, Italy). The neutrophil-to-lymphocyte ratio (NLR) was calculated as the neutrophil count/lymphocyte count (23–25), and the platelet-to-lymphocyte ratio (PLR) as the platelet count/lymphocyte count (25, 26). Reference ranges were defined as 4,800–10,800/µL for WBC, 1,800–7,800/µL for neutrophil count, 1,000–4,800/µL for lymphocyte count, 12–18 g/dL for hemoglobin concentration, 130,000–450,000/µL for platelet count, 7.4–10.4 fL for MPV, 0.4–4.8 µIU/mL for TSH concentration, 0.8–1.71 ng/dL for FT4 concentration, 0.6–1.6 ng/mL for TT3 concentration., <34IU/ml for anti-TPO, <115 IU/mL for anti-Tg, <0.3 mg/dL for CRP concentration, and <20 mm/hr for ESR.

### Statistical Analysis

Continuous variables in the two groups were compared using the Student’s *t*-tests or Mann–Whitney *U* test, and categorical variables in the two groups were compared using the χ^2^ test or Fisher’s exact test. Correlations between variables among multiple groups were assessed using one-way Analysis of Variance (ANOVA), two-way ANOVA, or mixed models. Factors significantly associated with the development of the hypothyroid phase were determined using a logistic regression analysis. All statistical analyses were performed using IBM SPSS Statistics, Version 27.0 (IBM Corp., Armonk, NY, USA), with p values <0.05 considered statistically significant.

## Results

### Baseline Demographic and Clinical Characteristics

The baseline demographic and clinical characteristics of the 37 patients with SAT and the 120 healthy controls are presented in **Table 1**. Sex distribution and age at diagnosis were similar between the two groups. Serum TSH (p <0.05), hemoglobin concentration (p<0.05), and MPV (p=0.016) were significantly higher in the control group than in the SAT group, whereas free T4 concentration (p<0.05), WBC count (p<0.05), neutrophil count (p<0.05), lymphocyte count (p=0.012), platelet count (p<0.05), NLR (p<0.05), and PLR (p=0.014) were significantly higher in the SAT group than in the control group. Median thyroid volumes were significantly greater in the SAT than in the control group (12.70 cm^3^ [range: 5.00–24.53 cm^3^] vs 8.87 cm^3^ [range: 4.15–23.55 cm^3^], p<0.05).

**Table 1:** Baseline demographic and clinical characteristics.

	Healthy controls (N = 120)	SAT patients (N = 37)	P value
Female sex (%)	94 (78.3%)	32 (86.5%)	0.349
Age (mean, ± SD)	50.58 ( ± 9.78)	51.57 ( ± 9.28)	0.589
TSH (µIU/mL)	1.90 (0.41–4.25)	0.01 (0.01–1.76)	<0.05
Free T4 (ng/dL)	1.20 (0.80–1.60)	2.18 (0.80–7.77)	<0.05
WBC (/µL)	5200 (2900–10400)	8300 (4900–26100)	<0.05
Neutrophil (/µL)	2910 (1460–8550)	5290 (2420–22940)	<0.05
Lymphocyte (/µL)	1895 (950–2990)	2200 (1100–3710)	0.012
Hemoglobin (g/dL)	14.33 ( ± 1.41)	12.41 ( ± 1.21)	<0.05
Platelet (× 10^3/^µL)	250 (142–496)	349 (213–533)	<0.05
NLR	1.62 (0.63–6.20)	2.43 (1.19–10.24)	<0.05
PLR	134.52 (54.41–408.00)	168.12 (77.02–391.82)	0.014
MPV (fL)	9.61 ( ± 1.02)	9.14 ( ± 1.00)	0.016
ESR (mm/hr)		53.30 ( ± 21.30)	N/A
CRP (mg/dL)		1.51 (0.19-8.12)	N/A
Thyroid volume (cm^3^)			
Initial	8.87 (4.15–23.55)	12.70 (5.00–24.53)	<0.05
3 months of follow-up	8.87 (4.15–23.55)	8.34 (4.08–22.78)	0.545

SAT, subacute thyroiditis; TSH, thyroid-stimulating hormone; free T4, free thyroxine; WBC, white blood cell count; NLR, neutrophil-to-lymphocyte ratio; PLR, platelet-to-lymphocyte ratio; MPV, mean platelet volume; ESR, erythrocyte sedimentation rate; CRP, C-reactive protein. N/A, Not Applicable.

### Subgroup Analysis Based on the Presence of Hypothyroid Phase

Patients with SAT were divided into those in a hypothyroid phase and those in a euthyroid phase during the course of the disease (**Table 2**). Sex distribution, age, TSH, free T4, total T3, anti-TPO positivity, anti-Tg positivity, WBC count, neutrophil count, hemoglobin level, platelet count, NLR, PLR, MPV, CRP, ESR, and follow-up periods were similar between the subgroups. The lymphocyte count (p=0.003) was significantly higher in the hypothyroid group than in the non-hypothyroid subgroup. Although not statistically significant, the proportion of patients with bilateral involvement on initial ultrasonography was higher in the hypothyroid subgroup (p=0.071). The proportion of patients with creeping patterns (hypoechoic lesions migrating from one thyroid lobe to the other) on follow-up ultrasonography (p=0.038) and the cumulative dose of prednisolone (p=0.011) were higher in the euthyroid group than in the hypothyroid group. Among the hypothyroid subgroups, two patients presented with persistent hypothyroidism requiring levothyroxine replacement therapy.

**Table 2:** Subgroup analysis based on the development of hypothyroid phase.

	Absent hypothyroid phase (N = 11)	Present hypothyroid phase (N = 26)	P value
Female sex (%)	10 (90.9%)	22 (84.6%)	1.000
Age at diagnosis	57.0 (42.0–75.0)	49.0 (31.0–72.0)	0.056
TSH (µIU/mL)	0.033 (0.005–1.760)	0.008 (0.005–0.640)	0.076
Free T4 (ng/dL)	1.94 (1.10–5.84)	2.29 (0.80–7.77)	0.282
Total T3 (ng/mL)	1.73 (1.15–3.40)	1.84 (0.91–6.14)	0.725
Anti-TPO positivity	0/7 (0.0%)	0/21 (0.0%)	N/A
Anti-Tg positivity	0/9 (0.0%)	3/22 (13.6%)	0.537
WBC (/µL)	7700 (4900–11700)	8400 (5100–26100)	0.371
Neutrophil (/µL)	5440 (2420–8340)	5150 (2570–22940)	0.713
Lymphocyte (/µL)	2040 (1100–2200)	2350 (1160–3710)	0.003
Hemoglobin (g/dL)	12.20 (11.00–13.90)	12.25 (10.40–17.30)	0.980
Platelet (× 10^3/^µL)	310 (213–431)	366 (215–533)	0.086
NLR	3.20 (1.19–6.02)	2.25 (1.25–10.24)	0.267
PLR	171.36 (120.10–391.82)	165.55 (77.02–285.39)	0.518
MPV (fL)	9.4 (7.4–10.1)	9.3 (7.1–10.9)	0.571
CRP (mg/dL)	1.57 (0.26–6.02)	1.39 (0.19–8.12)	0.559
ESR (mm/hr)	48.0 (22.0–92.0)	51.5 (27.0–108.0)	0.475
Bilateral involvement^1^	2 (18.2%)	14 (53.8%)	0.071
Creeping pattern^2^	6 (54.5%)	4 (15.4%)	0.038
Persistent hypothyroidism	0 (0.0%)	2 (7.7%)	1.000
Use of prednisolone	11 (100.0%)	26 (100.0%)	N/A
Cumulative dose (mg)	770.0 (245.0–2890.0)	463.8 (122.5–1700.0)	0.011
Follow-up period (months)	8 (3–24)	8 (3–38)	0.490

SAT, subacute thyroiditis; TSH, thyroid-stimulating hormone; Free T4, free thyroxine; Total T3, total triiodothyronine; Anti-TPO, anti-thyroid peroxidase antibody; Anti-Tg, anti-thyroglobulin antibody; WBC, white blood cell count; NLR, neutrophil-to-lymphocyte ratio; PLR, platelet-to-lymphocyte ratio; MPV, mean platelet volume; CRP, C-reactive protein; ESR, erythrocyte sedimentation rate. N/A, Not Applicable.

^1^The proportion of SAT patients who showed bilateral involvement on initial thyroid ultrasonography.

^2^The percentage of SAT patients who had migrating patterns on thyroid ultrasonography (From one side to the other side of the thyroid gland).

### Time-Dependent Changes in Thyroid Volumes in Patients With SAT

**Figure 1:**
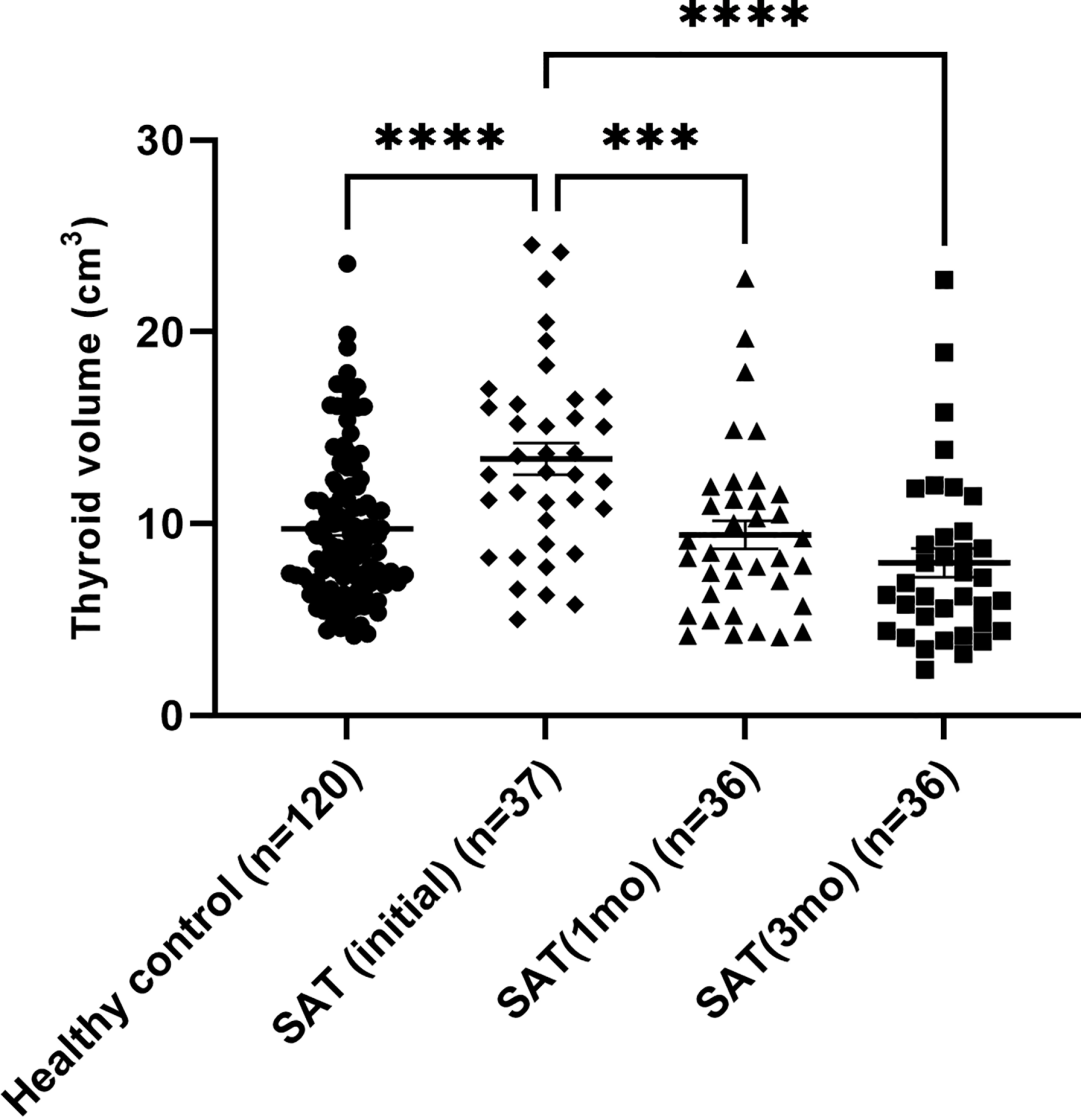
Time-varying thyroid volumes of subacute thyroiditis patients compared with those of healthy controls. ***P ≤ 0.001, ****P ≤ 0.0001.

**Figure 2:**
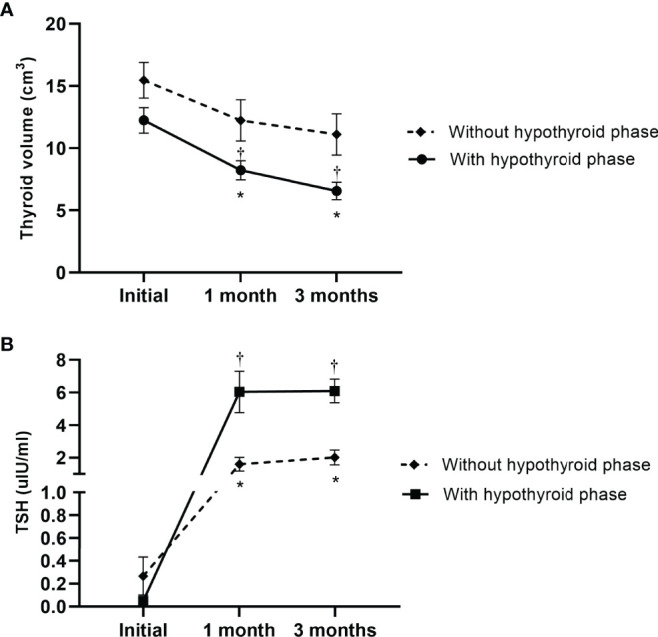
The change in **(A)** thyroid volumes and **(B)** serum TSH levels in the time course of subacute thyroiditis patients, who were divided based on the development of hypothyroid phase in their clinical courses. Asterisks represent significant difference between the two subgroups, and daggers imply significant differences in the initial features of each subgroup.

**Figure 1** shows a comparison of thyroid volumes over time in 37 SAT patients and 120 healthy controls. Initial thyroid volumes in the SAT group were larger than those of the control group (12.70 cm^3^ [range: 5.00–24.53 cm^3^] vs 8.87 cm^3^ [range: 4.15–23.55 cm^3^], p < 0.05). However, the thyroid volumes gradually became lower and smaller than those of the controls without significance at the 3 months’ follow-up visit (8.34 cm^3^ [range: 4.08–22.78 cm^3^] vs 8.87 cm^3^ [range: 4.15–23.55 cm^3^], p=0.545) (**Figure 1** and **Table 1**). The changes in thyroid volume and serum TSH levels between the two subgroups of SAT patients, who were divided based on the development of the hypothyroid phase, are shown in **Figure 2**. Two patients with persistent hypothyroidism were excluded from this analysis considering the possible effects of levothyroxine on thyroid volume. The initial thyroid volumes of the two subgroups were similar (p=0.113), but the thyroid volumes of the subgroup with the hypothyroid phase were significantly smaller than those of the subgroup without the hypothyroid phase at 1 month’s (p=0.025) and 3 months’ (p=0.006) follow-up visits (**Figure 2A** and **Supplementary Table S1**). When comparing the reduction rates of thyroid volume, the median reduction rate of thyroid volume was greater in patients in than not in a hypothyroid phase, especially during the first month (32.30% [range: 6.74-55.29%] vs. 19.41% [range: 5.75-51.76%], p=0.009) (**Supplementary Table S1**). The difference in serum TSH levels was initially not significant between the subgroups (p=0.071), but that in the hypothyroid subgroup became higher at the 1 month (p=0.006) and 3 month (p < 0.05) follow-up visits (**Supplementary Table S1**).

Case presentation of the patients who had persistent hypothyroidism after SAT episodes

Two women with persistent hypothyroidism following SAT episodes were included in this study. Both patients showed bilateral involvement on initial thyroid ultrasonography. After starting prednisolone, both patients showed a puffy face, edema, and fatigue with elevated TSH levels of 76.60 µIU/ml (patient 1) and 63.70 µIU/ml (patient 2). Both patients were prescribed 50µ of levothyroxine per day and medications were maintained throughout the follow-up period. The thyroid volumes decreased from 16.62 cm^3^ to 11.53 cm^3^ (patient 1, with a reduction rate of 30.61%) and from 11.11 cm^3^ to 4.97 cm^3^ (patient 2, with a reduction rate of 55.29%) at their 1 month follow-up visit.

### Univariate and Multivariate Analyses

Univariate and multivariate analyses were performed to evaluate the factors associated with the development of the hypothyroid phase in SAT (**Table 3**). Age, sex, initial TSH level, initial free T4 concentration, initial ESR level, bilateral involvement on initial thyroid ultrasonography, and reduction rate of thyroid volume within the first month were included. The reduction rate of thyroid volume within the first month of follow-up was the only significant factor in both the univariate (p=0.035) and multivariate (p=0.035) analyses.

**Table 3:** Predictive factors for hypothyroid phase in SAT.

	Univariate analysis		Multivariate analysis	
	OR (95% CI)	P-value	OR (95% CI)	P-value
Age	0.914 (0.835-1.002)	0.054		
Female sex	0.550 (0.054-5.570)	0.613		
Initial TSH	0.079 (0.002-2.628)	0.156		
Initial FT4	1.166 (0.657-2.068)	0.600		
Initial ESR	1.019 (0.982-1.058)	0.312		
Bilateral involvement on initial ultrasonography	5.250 (0.945-29.180)	0.058		
Reduction rate of thyroid volume within the first month^1^	1.084 (1.006-1.169)	0.035	1.084 (1.006-1.169)	0.035

SAT, subacute thyroiditis; WBC, white blood cell count; TSH, thyroid-stimulating hormone; free T4, free thyroxine; ESR, erythrocyte sedimentation rate.

^1^Reduction rate of thyroid volume within the first month was calculated as follows: (initial thyroid volume – thyroid volume measured at 1 month follow-up)/initial thyroid volume × 100 (%).

## Discussion

Previous reports have shown decreasing thyroid volumes after episodes of SAT and attempted to determine the association between ultrasonographic findings and the clinical course of SAT (19–21, 27). Gorges et al. reported a possible association between low residual thyroid volume and permanent hypothyroidism (27). It is noteworthy that a prospective study by Zhao et al. demonstrated a change in thyroid volumes of SAT patients that were initially larger than those of healthy controls and eventually became smaller, with the difference being greater in the hypothyroid patients at their 2 year follow-up visit (21). The present study also showed a similar trend, i.e., thyroid volumes were initially larger in SAT patients than in healthy controls, but subsequently decreased and eventually became smaller than those in healthy controls at the 3 month follow-up visit without significance. Furthermore, we compared thyroid volumes between the subgroups of patients with SAT, who were divided based on the presence of the hypothyroid phase, especially focusing on the acute phase in their clinical courses. In contrast to the similar initial thyroid volumes between the two subgroups, SAT patients in the hypothyroid phase showed significantly smaller thyroid volumes than patients not in a hypothyroid phase at the 1 month and 3 month follow-up visits. The reduction rates of thyroid volume within the first month were significantly different between the two subgroups, with univariate and multivariate analyses showing that this reduction rate was associated with the development of the hypothyroid phase after 1 month. These findings suggest that patients who experience greater thyroid volume reduction in their early phases will show greater depletion of thyroid hormone reserves and have a higher risk for developing a hypothyroid phase afterwards. These findings also suggest the potentially important role of serial thyroid ultrasonography examinations in better understanding the disease course of SAT.

In addition to the thyroid volume, the extent of involvement on thyroid ultrasonography has been assessed for its association with the prognosis of patients with SAT. A retrospective study found that ultrasonography patterns were not significantly associated with the risk for recurrence and permanent hypothyroidism (20). In contrast, bilateral involvement was reported to be more common in patients with hypothyroidism, suggesting that the extent of hypoechoic areas may be a potential marker for hypothyroidism during the course of SAT (28). Although it was not significant in the present study, the proportion of bilateral involvement on initial thyroid ultrasonography was higher in the hypothyroid phase group, and the two patients with persistent hypothyroidism included in this study were found to have bilateral hypoechoic lesions on their initial thyroid ultrasonography. However, we believe that a significant difference was not observed owing to the small number of patients.

Potential inflammatory markers, such as NLR, PLR, and MPV, have also been evaluated in patients with SAT (25, 29–34). A retrospective study that included 306 SAT patients and 102 controls showed that NLR and PLR were higher and MPV was lower in SAT patients, supporting SAT diagnosis (25). Another study reported that NLR and PLR were higher in patients with SAT than in patients with other causes of thyrotoxicosis, suggesting that these markers may be indicative of SAT (35). In agreement with previous reports, the present study showed higher NLR and PLR and lower MPV in SAT patients, supporting their potential as practical markers for SAT diagnosis, especially in resource-limited settings.

The treatment modality was found to be related to the clinical outcomes in SAT patients. The incidence of thyroid dysfunction was reported to be lower in patients treated with prednisolone than in those treated with NSAIDs or untreated patients (28). Steroids have shown better clinical outcomes than NSAIDs, especially in SAT patients positive for anti-TPO Ab, high ESR, and CRP (13). Unfortunately, we could not determine any relationship between the treatment modalities and thyroid function as all patients included in the current study were treated with corticosteroids. Previous reports have shown controversial results regarding the association between the corticosteroid dose and development of hypothyroidism. Gorges et al. reported that higher cumulative doses of prednisolone and female sex were associated with an increased risk for hypothyroidism in patients (27). However, they also mentioned that the results could have been affected by the severity of the episodes rather than the higher cumulative dose of steroids itself. A retrospective study by Hepsen et al. comparing low-and high-dose steroid treatments (48 mg vs. 16 mg of methylprednisolone) for SAT showed that the cumulative dose of methylprednisolone and female sex were similar between subgroups, which were divided based on the development of permanent and transient hypothyroidism, supporting the usefulness of low-dose steroid treatment (36). In our study, female sex showed no significant difference between the two subgroups, and the cumulative dose of prednisolone was higher in the euthyroid group than in the hypothyroid phase group. A possible explanation for this finding could be the higher proportion of creeping patterns on ultrasonography in patients without transient hypothyroidism, resulting in longer treatment periods.

The present study has several limitations. Due to the retrospective design and small sample size, it was difficult to propose a monitoring scheme to predict the risk for developing permanent hypothyroidism in the future. Through further studies, we believe that we can propose a more detailed and promising monitoring scheme for predicting a subsequent hypothyroid phase, which can possibly progress to persistent hypothyroidism. In addition, anti-thyroid antibodies were not assessed in all SAT patients, as they are not essential for the diagnosis of SAT, but some patients may be positive for these antibodies (37, 38). This prevented an evaluation of the effects of these antibodies on abnormal thyroid function or thyroid volume reduction. Another limitation was the lack of data on clinical characteristics (including thyroid function tests, anti-thyroid antibodies, and the size of the thyroid gland) of the patients before their SAT episodes; this information may be helpful for better understanding the clinical course of SAT. Moreover, as all SAT patients enrolled in this study were treated with corticosteroids, we could not compare their clinical course with other treatment methods (NSAIDs). Furthermore, as only two patients had persistent hypothyroidism, we could not directly evaluate the association between thyroid volume and persistent thyroid dysfunction. Additional studies with larger sample sizes and an increased proportion of patients with persistent hypothyroidism are needed.

In conclusion, SAT patients show decreasing thyroid volumes in their clinical courses, and patients with preceding greater reduction of thyroid volume in their acute phases (especially within 1 month) tend to have a higher chance of developing the hypothyroid phase, which can lead to persistent hypothyroidism. Additionally, we conclude that serial thyroid ultrasonography examinations may help understand the clinical course of SAT.

## Data Availability Statement

The raw data supporting the conclusions of this article will be made available by the authors, without undue reservation.

## Ethics Statement

The studies involving human participants were reviewed and approved by the Institutional Review Board of Chonnam National University Hwasun Hospital, Hwasun, South Korea. Written informed consent for participation was not required for this study in accordance with the national legislation and the institutional requirements.

## Author Contributions

JP: Conceptualization, data curation, manuscript drafting, and manuscript revision. WC: Conceptualization, data curation, and revision of the manuscript. AH: Study design, data interpretation, and manuscript revision. JY: Study design, data interpretation, and manuscript revision. HK: Conceptualized, interpreted the data, and revised the manuscript. H-CK: Conceptualization, supervision, data interpretation, manuscript drafting, and manuscript revision. All authors contributed to the article and approved the submitted version.

## Conflict of Interest

The authors declare that the research was conducted in the absence of any commercial or financial relationships that could be construed as a potential conflict of interest.

## Publisher’s Note

All claims expressed in this article are solely those of the authors and do not necessarily represent those of their affiliated organizations, or those of the publisher, the editors and the reviewers. Any product that may be evaluated in this article, or claim that may be made by its manufacturer, is not guaranteed or endorsed by the publisher.
