# Supplementary material for: Early Thyroid Volume Reduction in Subacute Thyroiditis Can be a Potential Indicator for Hypothyroidism
Source: Front Endocrinol (Lausanne). 2022 May 30;13:888018. doi: 10.3389/fendo.2022.888018 (PMC9196237; doi:10.3389/fendo.2022.888018)
Supplement: Supplementary file 1 [file Table_1.docx]

| **Supplementary table 1. Thyroid volumes and serum TSH levels during the clinical course in subacute thyroiditis patients** | | | |
| --- | --- | --- | --- |
| Thyroid volume (cm^3^) | Absent hypothyroid phase (N=11) | Present hypothyroid phase (N=24) | P value |
| Initial | 15.08 (10.20-24.17) | 12.38 (5.00-24.53) | 0.113 |
| 1 month | 9.76 (7.80-22.78) | 7.24 (4.08-17.92) | 0.025 |
| 3 months | 8.71 (5.80-22.71) | 5.73 (2.40-15.80) | 0.006 |
| Reduction rate of thyroid volume (%)^1^ | | | |
| Initial – 1 month | 19.41 (5.75-51.76) | 32.30 (6.74-55.29) | 0.009 |
| Initial – 3 months | 24.04 (6.04-63.55) | 46.33 (22.65-71.61) | 0.003 |
| 1 month – 3 months | 11.25 (0.31-25.67) | 19.27 (2.75-45.05) | 0.133 |
| TSH level (uIU/ml) |  |  |  |
| Initial | 0.033 (0.005-1.760) | 0.007 (0.005-0.640) | 0.071 |
| 1 month | 1.690 (0.005-4.290) | 5.690 (0.019-24.870) | 0.006 |
| 3 months | 2.270 (0.010-4.310) | 4.720 (0.010-12.610) | <0.05 |

TSH; Thyroid stimulating hormone.

^1^The reduction rate of thyroid volume was calculated as follows: [ (Thyroid volume measured at earlier visit – thyroid volume measured at later visit) / thyroid volume measured at former visit ] x 100 (%).
